# Understanding Public Acceptance of Autonomous Vehicles Using the Theory of Planned Behaviour

**DOI:** 10.3390/ijerph17124419

**Published:** 2020-06-19

**Authors:** Kum Fai Yuen, Grace Chua, Xueqin Wang, Fei Ma, Kevin X. Li

**Affiliations:** 1 School of Civil and Environmental Engineering, Nanyang Technological University, Singapore 639798, Singapore; kumfai.yuen@ntu.edu.sg (K.F.Y.); chua0873@e.ntu.edu.sg (G.C.); 2 Department of International Logistics, Chung-Ang University, Seoul 06974, Korea; 3 School of Economics and Management, Chang’An University, Xi’an 710064, China; mafeixa@chd.edu.cn; 4 Ocean College, Zhejiang University, Hangzhou 310058, China; kxli@zju.edu.cn

**Keywords:** autonomous vehicles, theory of planned behaviour, structural equation modelling, intention, acceptance

## Abstract

Public acceptance of autonomous vehicles (AVs) is vital for a society to reap their intended benefits such as reduced traffic accidents, land usage, congestion and environmental pollution. The purpose of this paper is to use the theory of planned behaviour to pinpoint and examine the components affecting public acceptance of AVs. A model consisting of a network of hypothesised relationships is introduced. Thereafter, 526 residents in Seoul, Korea, were given a survey created for this research. Subsequently, to evaluate the collected information and estimate the model, structural equation modelling was adopted. The outcomes show individuals’ mindset on AVs, subjective customs, and behavioural influence directly influencing the acceptance of AVs. Furthermore, cognitive and emotive factors, namely comparative advantage, compatibility, complexity and hedonic motivation indirectly influence the acceptance of AVs via mindset and behavioural manipulation. Based on analysing the cumulative effect, attitude emerged with the strongest effect on public acceptance of autonomous vehicles. After this is, in decreasing order of influence, behavioural control, relative advantage, subjective norms, compatibility, hedonic motivation and complexity. The findings of this study implicate the prioritisation and allocation of resources, and policies relating to marketing, education, subsidisation and infrastructure development to better public acceptance of AVs.

## 1. Introduction

Autonomous vehicles (AVs) are self-driving vehicles capable of navigating roads, parking, sensing the environment and planning routes autonomously with minimum or without human conduction. With the rapid increase in trials and progress of the technological readiness of AVs and their infrastructural ecosystem, for example, the fifth-generation cellular network technology, it is reported that AVs would be launched within the next decade [1]. 

The benefits provided by AVs can be enjoyed by many stakeholders [2]. From the users’ perspective, AVs are expected to improve safety by eliminating human errors caused by misjudgement, fatigue, impaired driving and environmental influences [3]. AVs are also noted to improve users’ experience by providing more comfortable rides due to smoother braking and acceleration [4]. Further, they free up users’ time which is originally dedicated to driving, allowing them to participate in other pertinent activities while commuting to their destination. From a societal perspective, AVs encourage vehicle sharing which reduces the demand for a private car. Also, AVs are noted to be more energy-efficient than convention vehicles. These decrease congestion, pollution and free up parking space for commercial or recreational use. From the car manufacturers’ perspective, their ability to launch AVs would demonstrate the advancement of their research and development capabilities, which has a beneficial impact on their brand. Moreover, the launch of AVs would confer them a first-mover advantage and opportunities to expand their market shares by catering to early adopters of new innovations or individuals who are incapable of driving such as senior citizens, people with medical or mobility conditions or those concerned with or interested in impaired driving [5].

Despite the various benefits that AVs can offer to stakeholders, such utility can only be realised or maximised if critical mass in the use of AVs is achieved. For the launch of any new technologies such as AVs, previous studies have shown that critical mass can only be attained if there is widespread public acceptance [6]. In this context, acceptance is defined as an individual’s assent or approval for the usage of AVs. Since humans are habitual creatures, the introduction of new technologies such as AV is expected to present huge public resistance to the acceptance of AVs. This observation is expected because AVs are a significant technological improvement from traditional vehicles. Consequently, using AVs may require training creating inconvenience and resistance, and raise concerns over users’ faith in AVs’ capability to execute their tasks securely and dependably [7].

Given the importance of garnering public acceptance towards AVs, much research has been undertaken to examine the components affecting public acceptance of AVs [8,9]. The existing research can be classified into two streams. The first stream of research has received the most attention [3]. It analyses the demographics and travel characteristics of individuals that could influence public acceptance of AVs. For example, Liljamo, et al. [10] found that individuals who were male, received more education, lived in densely populated areas are more receptive to AVs. The second stream of research, which has received relatively less attention, has focused on identifying latent psychological or behavioural constructs that influence public acceptance of AVs [11,12]. Within this stream of research, some studies have introduced theories to examine the public acceptance of AVs [13]. The most common theory that has been introduced is the technology acceptance model and/or its extensions, incorporating trust or risk concepts [6,14,15,16,17]. 

Although previous theoretical studies have enhanced understanding of public acceptance of AVs, relatively fewer studies have analysed the importance of attitude’s impact on public acceptance [5,8,18]. In social psychology, attitude is an individual’s cognitive and affective assessment of an object, which in this context, refers to AVs. The evaluation can be negative or positive. Haboucha, Ishaq and Shiftan [18] and Hudson, et al. [19] found that the general attitude of individuals is a strong determinant of acceptance of AVs as compared to other latent or demographic variables. Their results have implied the importance of incorporating attitude to model public acceptance of AVs.

To tackle the abovementioned gaps in research, the study’s objective is to extend existing theoretical research on public acceptance of AVs by using the theory of planned behaviour (TPB). TPB’s effectiveness in explaining behaviour is widely acknowledged in many applications, including recent transport innovations involving automated parcel delivery, drone delivery, ride-hailing services, electric vehicles and vehicle sharing [20,21,22,23]. Therefore, TPB is an appropriate theoretical lens to be applied to explain the public acceptance of AVs. With this in mind, the theory suggests that individuals’ tendency to accept AVs is not only determined by their (1) attitude towards AVs, but also the (2) subjective norms and (3) perceived control over their decision to accept AVs [24,25]. Attitude and perceived control are in turn influenced by individuals’ ideas, beliefs, values and perceptions which can vary with a specific context. Hence, another key contribution of this study is that it reviews and identifies pertinent ideas, beliefs, values and perceptions that are specific to the AV context. 

The rest of this paper is organised in the following manner. Firstly, anchored on TPB, this paper proposes and specifies a model comprising of a network of hypotheses to explain the public acceptance of AVs. Subsequently, it elaborates on the survey design and administration which are conducted on residents in Seoul, Korea. Thereafter, this study describes the data analysis method (i.e., structural equation modelling) used to estimate the model. It then presents the results and discussion. Lastly, this study concludes by discussing the theoretical and policy implications, limitations and recommendations for future studies.

## 2. Theory, Model and Hypotheses

**Figure 1:**
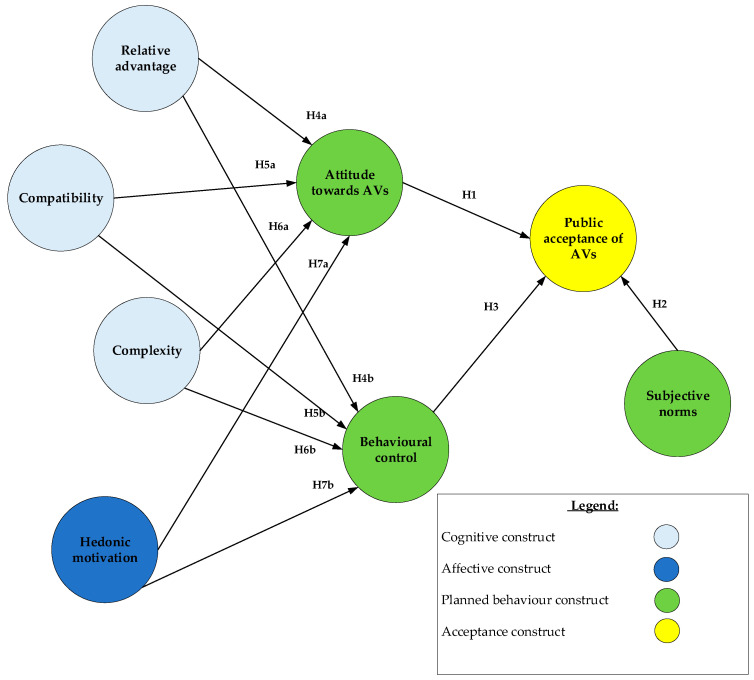
The theoretical model.

Figure 1 shows the model proposed by this study to explain the public acceptance of AVs. In essence, this research paper aims to explain the public acceptance of AVs using TPB. 

The theory holds that human behaviour is mainly guided by three psychological dimensions. They are individuals’ attitudes toward AVs, subjective norms and behavioural control. Each of these dimensions can explain the public acceptance of AVs from a different perspective. The subsequent subsections review the contribution of each dimension. Thereafter, this section discusses the factors influencing the three dimensions.

### 2.1. The Influence of Attitude on Public Acceptance 

Attitude is the mind’s inclination or preference, manner, disposition, emotion and stance towards a human or object. In this context, individuals’ attitude towards AVs concerns their personal evaluation of AVs, which could be positive or negative [26]. Previous research has shown that possessing a positive attitude towards an object or technology would directly result in their acceptance or usage [27]. Likewise, possessing a negative attitude would result in rejection or discontinuation of using the object or technology. 

A few studies have focused on analysing the attitude of the public towards AVs. It has been reported that certain individuals’ profiles related to gender (i.e., male), education (i.e., high), age (i.e., young), and location of residence (i.e., urban) are positively correlated with their attitude towards AVs [10]. Further, it has been found that possessing a pro-AV attitude is the strongest predictor of public acceptance as compared to demographic variables (e.g., income, age, gender and education) as well as other latent variables (e.g., technology interest, environmental concern and driving enjoyment) [5,18]. The finding stresses the significance of attitude in influencing public acceptance of AVs. Hence, the subsequent hypothesis is presented.

**Hypothesis** **1 (H1).** *Possessing a positive attitude towards AVs has a positive influence on public acceptance of AVs.*

### 2.2. The Influence of Subjective Norms on Public Acceptance 

According to TPB, acceptance of AVs is not solely based on an individual’s assessment of AVs (i.e., attitude). Individuals are social creatures who will also take references from their significant referents such as friends, colleagues and media in their decision making. Therefore, social influence, which is reflected by the expectations of an individual’s significant references about using AVs plays a significant role in influencing public acceptance of AVs. Recent research in AVs supports this relationship [6,15,28].

Subjective norms, which is a dimension proposed by TPB to influence public acceptance of AVs, can be viewed as an extension of social influence. The tenet of subjective norms suggests that in addition to social influence or pressure, an individual’s motivation to comply with the pressure from his or her significant referents is equally important to explain the acceptance of AVs [29]. Therefore, the introduction of subjective norms to explain the acceptance of AVs can be view as a construct that expands previous AV research which only focuses on the effects of social influence exerted by an individual’s significant referents. Based on the above discussion, the following hypothesis is developed.

**Hypothesis** **2 (H2).** *Subjective norms have a positive influence on public acceptance of AVs.*

### 2.3. The Influence of Behavioural Control on Public Acceptance 

Lastly, apart from individuals’ attitudes and subjective norms, their belief in successfully overcoming the perceived difficulties in learning and using AVs also plays a crucial role in accepting AVs. This is reflected by behavioural control which is the third dimension of TPB. 

In this context, behavioural control refers to the presence or absence of control factors (i.e., facilitators and barriers) that enable or prevent the adoption of AVs. Under behavioural control, Talebian and Mishra [7] introduced the concept of resistance and proposed that it is fundamental to the adoption or acceptance of AVs. The authors proposed that resistances can be segmented into two groups: Functional and psychological barriers. Functional barriers are aspects related to individuals’ current workflow and habits whereas psychological barriers are dimensions related to individuals’ prior beliefs. Some examples related to functional barriers are AVs’ usage patterns, their utility, and uncertainty risks whereas those related to psychological barriers may include traditions and norms, and perception barrier. Similarly, Fagnant and Kockelman [4] identified several functional barriers that inhibit the adoption of AVs. They are mainly functional barriers which include (1) vehicle costs, (2) AV certification, (3) litigation, liability, and perception, (4) security and (5) privacy. 

TPB suggests that behavioural control connotes two concepts: Facilitating conditions and self-efficacy [30]. The aforementioned studies have mainly focused on analysing the facilitating conditions which refer to supporting resources (i.e., time, money, effort, knowledge, skills, supporting infrastructure and media or government persuasion) that motivate the adoption of AVs. For instance, addressing the barriers identified by previous studies (e.g., reduced vehicle cost and increased privacy) can reduce the number of resources required by individuals to purchase and use AVs and hence, motivates the adoption of AVs. Similarly, ensuring greater integration between AVs and existing transport infrastructures and networks can result in the adoption of AVs due to more supporting resources perceived by the public.

Self-efficacy, which has not been adequately studied by the literature on AVs, is another component of behavioural control [31]. It can be viewed as a psychological facilitator which refers to possessing the confidence or belief to acquire or dedicate the necessary resources (i.e., knowledge, time and effort) to enquire, learn and use AVs [3]. Due to the perceptual nature regarding individuals’ assessment of the facilitating condition, the self-efficacy of an individual to confidently use AVs is crucial. 

Based on the above argument, this research proposes that the public is more likely to accept AVs if they perceive more supporting resources and possess strong self-efficacy. Therefore, the subsequent hypothesis is presented.

**Hypothesis** **3 (H3).** *Behavioural control has a positive influence on public acceptance of AVs.*

### 2.4. The Application of Decomposed TPB

To offer greater insights into the acceptance or usage of an object or innovation, some studies have analysed the factors influencing the three dimensions posited by TPB which include attitude, subjective norms and behavioural control [23,32]. This process is also termed “decomposition” in the literature. Within the TPB framework, it has been proposed that individuals’ ideas, beliefs, values, and perceptions influence the three dimensions of TPB. Notably, these ideas, beliefs, values, and perceptions are noted to vary according to context (i.e., the object or innovation of interest). 

Drawing on principles from social psychology (i.e., attitude formation theory), this study introduces cognitive and affective components of AVs which could influence the dimensions of TPB. The cognitive component refers to individuals’ beliefs (i.e., evaluation) about specific attributes of AVs whereas the affective component refers to individuals’ emotions or feelings about specific attributes of AVs. 

Specifically, this study proposes that the cognitive and affective components associated with AVs have direct influences on public attitude. Since attitude relates to individuals’ overall assessment of AVs, their attitude towards AVs would be positive if the outcome of their cognitive and affective assessment of AVs are favourable. Similarly, this study also proposes that the cognitive and affective components would directly influence behavioural control. Positive assessment of the components, in particular, affective components, could serve as facilitators that motivate individuals’ control of using AVs and hence encourage their acceptance. However, this study proposes no direct relationship between the components and subjective norms because the latter mainly concerns social influence. Therefore, it is logical to expect that the cognitive or affective component is not related to subjective norms.

#### 2.4.1. The Influence of Cognitive Attributes on Attitude and Behavioural Control

This study employs the innovation diffusion theory to identify the cognitive attributes associated with AVs. According to the theory, the spread in the adoption of innovation within a society is influenced by five cognitive attributes which are related to the relative advantage, compatibility, complexity, trialability and observability of an innovation. The theory is a suitable theoretical lens because an AV is considered an innovation that will revolutionise existing transport systems, processes and practices. Since AVs are still under development and at the trial stage, the trialability and observability of AVs are very limited. Hence, they are excluded from the review.

Relative advantage is the extent to which AVs are viewed to be superior to conventional vehicles [13]. For example, AVs are reportedly safer than traditional vehicles because navigation and sensing tasks are performed by technologies which are more reliable than human senses [11]. In addition, AVs can confer fuel savings due to their ability to optimise routes and execute smoother braking and acceleration [4]. As a result of the above functionalities, AVs are also able to offer users greater comfort and shorter commuting time [33]. Lastly, AVs give their owners potential income-earning prospects; for instance, AVs can be engaged to provide ride-hailing services when unused. 

In general, the aforementioned advantages of AVs would have a positive effect on the attitude of individuals. In addition, the advantages would also act as facilitators that ease the public’s resistance against the adoption of AVs. Therefore, the following hypotheses are proposed.

**Hypothesis** **4a (H4a).** *Relative advantage has a positive influence on attitude towards AVs.*

**Hypothesis** **4b (H4b).** *Relative advantage has a positive influence on behavioural control.*

Compatibility is the extent to which AVs are viewed as per existing ideals, way of life, traditions, and public transportation demands [20]. 

In general, using AVs aligns with travel behaviour in urban cities which is more mobile. AVs are more compatible with individuals adopting a mobile lifestyle because of AVs’ self-parking capability. This facilitates trip-chaining [34]. Consequently, AV users enjoy time-savings as they can get off at their destinations upon arrival instead of having to hunt for parking areas or wait for vehicle parking space. The self-park function can also potentially save costs for users because AVs can self-park in less expensive parking areas [4]. Furthermore, with a greater proportion of the population commuting to work or study, transport needs have evolved from single to multiple drop-off locations. AVs would be well-suited for this purpose because of their ability to self-drive and park. In addition, AVs are better suited for the growing demand for shared driving or ownership [35], which can reduce potentially further reduce costs [36].

Besides, AVs can be a cleaner substitute for conventional vehicles [37], by minimising air pollution and traffic congestion. With and the society’s growing concern over urban sustainability, AVs are more in sync with evolving social values. Therefore, based on the above discussions, the following hypotheses are proposed.

**Hypothesis** **5a (H5a).** *Compatibility has a positive influence on attitude towards AVs.*

**Hypothesis** **5b (H5b).** *Compatibility has a positive influence on behavioural control.*

Complexity is the extent to which society perceives AVs to be tough to comprehend or utilise. [27,38]. 

According to Miyazaki and Kijima [39], complexity is influenced by an individual’s interaction with the AV’s interface. The greater is the number of interfaces, the more complex AV is perceived to be. Further, complexity is influenced by the individual’s interest, competency, and beliefs about AVs. Essentially, if AVs were viewed to be difficult to use, they would have a negative influence on the public’s attitude towards AVs. Further, complexity would act as a functional barrier that reduces behavioural control because more resources are needed for individuals to learning AVs which creates resistance. Hence, the following hypotheses are proposed.

**Hypothesis** **6a (H6a).** *Complexity has a negative influence on attitude towards AVs.*

**Hypothesis** **6b (H6b).** *Complexity has a negative influence on behavioural control.*

#### 2.4.2. The Influence of Affective Attributes on Attitude and Behavioural Control

Emotion or affect is an area that is understudied by existing innovation and technology acceptance research. This study proposes hedonic motivation as an attribute to represent the affective component of AVs. Hedonic motivation refers to the amount of pleasure that is derived from using AVs. On the one hand, the notion that AV is an innovation may create positive emotions such as fun and excitement, particularly for those who are tech-savvy or risk-seeking [3]. On the other hand, it may create negative emotions such as anxiety and fear for laggards or those who are risk-averse. 

Although the novelty of AVs will erode over time and hence diminishing its emotional utility, positive emotions can be generated by focusing on the design of AVs such as rapid acceleration, internal and external aesthetics of the vehicle, an artful display of the information on the dashboard and technological sophistication [23]. Further, Nordhoff, et al. [40] suggested the continual incorporation of “wow” factors in the utilisation of space in AVs, customised to users’ trip characteristics and preference. For instance, a business, social or recreational function such as internet cafes, conference rooms or social networking can be incorporated in the design of AV. This would make travelling on AVs enjoyable or fun as users commute to their desired destinations. Possessing positive emotions about adopting AVs would have positive influences on the attitude and improve self-efficacy (i.e., behavioural control) of individuals. Therefore, the following hypotheses are proposed.

**Hypothesis** **7a (H7a).** *Hedonic motivation has a positive influence on attitude towards AVs.*

**Hypothesis** **7b (H7b).** *Hedonic motivation has a positive influence on behavioural control.*

## 3. Method

The objective of this study is to examine the factors influencing public acceptance of AVs through the theoretical lens of TPB. This study proposes that public acceptance of AVs is affected by the attitude, subjective norms and behavioural control of individuals. The attitude and behavioural control of individuals are in turn influenced by cognitive and affective attributes which include relative advantage, compatibility, complexity and hedonic motivation. The subsequent subsection discusses the indicator selection, survey design, and administration procedures and demographics of the survey respondents.

### 3.1. Indicator Selection

Since the variables of interest are latent (i.e., constructs), indicators are selected from the literature to operationalise each variable. The constructs include attitude, subjective norms, behavioural control, relative advantage, compatibility, complexity, and acceptance of AVs. Table 1 shows the constructs, indicators, and the research studies used to develop the indicators.

### 3.2. Survey Design and Administration

A survey questionnaire is designed to collect information from the public. It is divided into three sections. The first section provides brief background information about AVs and the purpose of the study. It also defines the level of automation in AVs which is the Society of Automotive Engineers (SAE) 4 and above. This indicates that users do not need to control the AV for navigation and acceleration, monitor the environment or perform an ad-hoc intervention. The second section contains demographic questions such as the respondents’ gender, age, income, education, driving experience and vehicle ownership. Finally, in the third section, it comprises the indicators presented in Table 1. 

The questionnaire was translated to Korean by a professional editor and administered to residents in Seoul, Korea. Only Korean citizens were considered so that their statistics can be compared with the population of Korea. The survey was administered concurrently at five Seoul subway stations exits with significant passenger movement. Randomly chosen, these sites consist of Sinchon, Apgujeong, Seoul, Suwon and Express Bus Terminal stations. At each station, four student helpers were assigned to randomly engage passengers in completing the survey. These selected passengers were first questioned for screening purposes—to find out if they were residing in Seoul and willing to participate in the survey. If they meet the screening requirements, they proceed to answer the questionnaire online or on hardcopy. Both the English and Korean versions were available for the respondents. All participants are rewarded with a cash voucher. For seven consecutive days, the survey was executed from 8 am to 8 pm in April 2019. 

In total, there were 723 completed survey questionnaires. After rejecting the invalid ones, 526 questionnaires were utilized for further study—242 were done in softcopy and 284 were done in hardcopy.

### 3.3. Demographics of Respondents

Table 2 states the 526 respondents’ profile. With 48% of the respondents being male and 52% of the respondents being female, the gender ratio was equally distributed. The sample’s gender ratio is similar to the populace’s gender ratio of 50.1% male and 49.9% female.

Secondly, nearly half of the sample received a yearly income of 10–40 million KRW (42%) and possessed a vehicle (49%). Minimally greater than the population’s average vehicle ownership value of 0.45, the sample’s average vehicle ownership is 0.73. This disparity is because the survey was administered in Seoul, South Korea’s capital city. Consequently, the sample’s vehicle ownership average is understandably greater than the populace’s average.

Furthermore, 71% of the respondents are minimally a bachelor’s degree graduate. The conclusion that most of the respondents are well-educated is aligned with the importance Korea places on education. With 51.5 million people in Korea, 70% of their 24- to 35-year-olds are reported to have finished university education in 2018. Additionally, a higher percentage of highly educated citizens are expected to reside in Seoul, where the survey was administered, relative to other Korean cities. Therefore, it is within the expectation that 71% of the respondents possess a bachelor’s degree. This offers validation for the sample’s representativeness.

## 4. Results and Discussion

As this paper evaluates the associations between latent constructs, structural equation modelling is adopted to study the obtained survey information. The approach comprises two stages that are to be conducted sequentially. The first stage involves conducting a measurement model analysis (i.e., confirmatory factor analysis) to determine the validity and reliability of the indicators used to operationalise the constructs. The second stage involves conducting a structural model analysis whereby the relationships between the constructs (i.e., hypotheses) are estimated. Finally, since the proposed model (Figure 1) involves mediation, a third stage is introduced whereby a bootstrapping technique is conducted to analyse the magnitude and significance of the direct, indirect and total effects of the constructs. The analyses are conducted using AMOS 19.0 (IBM, New York, USA).

### 4.1. Measurement Model Analysis

The first stage of the analysis involves conducting a confirmatory factor analysis to ascertain the model fit, reliability, and validity of the indicators used to operationalise the constructs.

Table 3 shows the confirmatory factor analysis results. It presents the overall model fit, the standardised factor loadings of the indicators (λ), the average variance extracted (AVE) and composite reliability (CR) of the constructs.

On the whole, the model fit indices shown in Table 3 show that the measurement model possesses a good fit. The fit indices meet the requirements of Hu and Bentler [46], which suggests a good model fit.

Regarding the reliability of the indicators, the λ of the indicators and CRs of the constructs are analysed. Table 3 indicates that the λ for all indicators are above the desired value of 0.80. This shows that a greater proportion of the variances of the indicators is described by the assigned construct instead of the errors. Next, the CRs of the constructs are above 0.80 which further reinforces the above assessment [47].

The indicators’ validity was examined with the use of convergent and discriminant validity. A table which shows the AVEs on the main diagonal, correlation below the main diagonal and squared correlation above the main diagonal is presented in Table 4. Firstly, convergent validity is confirmed because the AVE is greater than 0.50 [48]. Furthermore, discriminant validity is ascertained because the AVE a construct is greater than its squared correlations with other constructs. This shows that the indicators correlate more with the loaded construct rather than with other constructs. 

### 4.2. Structural Model Analysis

**Figure 2:**
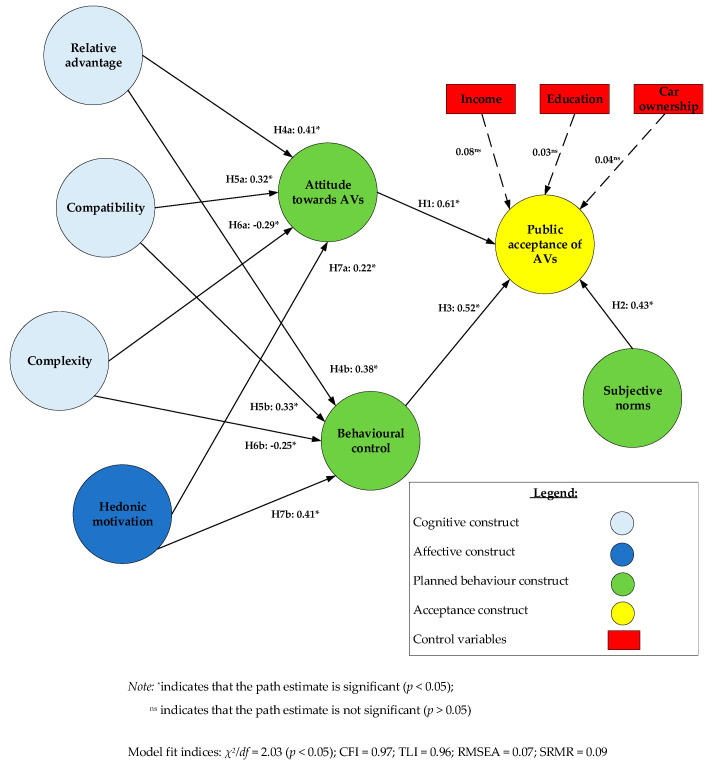
Parameter estimation of the proposed model.

Figure 2 indicates the estimated structural parameters of the proposed model. Further, control variables comprising income, education, and car ownership were incorporated into the model to take responsibility for their influence on public acceptance of AVs. These factors were observed to affect the uptake of AVs considerably [18,49].

As shown in Figure 2, public acceptance of AVs is regressed on three control variables—“income“, “education“ and “car ownership”. The effects of the control variables are not significant. This finding contradicts the literature which proposes that individuals with greater income are more inclined to accept AVs, which are expected to be more expensive than conventional vehicles. Those more educated are more aware of the benefits of AVs and hence, possess a greater tendency to accept AVs. In addition, individuals who own a vehicle might have greater acceptance towards AV than those who do not own a vehicle because of the possibility of replacing their existing vehicle. Nevertheless, the insignificant results suggest the presence of stronger contributing factors of public acceptance of AVs (i.e., attitude, behavioural control and subjective norms). To some extent, the results corroborate previous studies which found that latent variables are better predictors of public acceptance of AVs than sociodemographic variables [10,28]. 

All three TPB constructs which include attitude toward AVs, subjective norms and behavioural control are noted to have a large, positive impact on public acceptance of AVs (*p* < 0.05). Hence, H_1_, H_2_ and H_3_ are accepted. Accordingly, the estimated standardised effects are 0.61, 0.43 and 0.52. In conjunction with the control variables, the TPB constructs explain approximately 74% of the variance in public acceptance of AVs (R^2^ = 0.74) which is considered high. This highlights the efficacy of TPB. The results indicate that possessing positive cognitive and affective assessment of AVs (i.e., attitude), expectations of significant referents as well as motivations to comply with their expectations of using AVs (i.e., subjective norms), and elimination of functional or psychological barriers (i.e., behavioural control) such as reducing AV price, overcoming the steep learning curve, addressing liability and privacy concerns and providing more supporting resources for the public to use AVs are the key determinants influencing the acceptance of AVs. 

Regarding the determinants of the three TPB constructs, it can be seen that the proposed cognitive and affective components have a significant influence on attitude and behavioural control. Therefore, the remaining hypotheses are accepted (i.e., H_4a_, H_4b_, H_5a_, H_5b_, H_6a_, H_6b_, H_7a_ and H_7b_). The cognitive components are represented by relative advantage, compatibility and complexity which are constructs obtained from the innovation diffusion framework [50]. The affective component is reflected by hedonic motivation. Accordingly, these components explain for a moderate amount of variance in attitude (R^2^ = 0.65) and behavioural control (R^2^ = 0.69).

For instance, AVs are relatively more advantageous than conventional vehicles in areas such as safety, comfort, transit-time, opportunities for mobility while impaired and fuel consumption. These advantages can positively contribute to the evaluation of AVs and hence, the attitude of individuals (i.e., H_4a_). Similarly, the advantages can serve as facilitators that motivate the use of AVs, which improves the behavioural control of individuals using AVs (i.e., H_4b_). 

Further, AVs are arguably more compatible with today’s values, lifestyle, historical lessons, and public’s transportation requirements, thus giving more convenience to individuals enjoying a mobile lifestyle involving trip-chaining, multiple pick-ups and drop-off locations. Additionally, AVs are more suited to satisfy the increasing public demand for greener transport. In this regard, greater compatibility has a positive effect on individuals’ evaluation of AVs and hence, their attitude (i.e., H_5a_). Similarly, greater compatibility creates convenience which serves as a facilitator that reduces the perceived resources required to use AVs and hence, improves behavioural control (i.e., H_5b_). 

Minimising the complexity of AVs may improve positive evaluation of AVs, improving individuals’ attitude of AVs (i.e., H_6a_). Similarly, reducing complexity serves as a facilitator that reduces the resources needed to comprehend the procedure of using AVs. This will create convenience which will improve individuals’ behavioural control with regards to using or accepting AVs (i.e., H_6b_).

Finally, concerning hedonic motivation, individuals who perceive using AVs as fun and entertaining would have a more positive evaluation of AVs, leading to an improved attitude towards AVs (i.e., H_7a_). Further, possessing a positive effect on AVs can improve the confidence and ability of individuals to learn, understand, and use AVs (i.e., self-efficacy) which is an integral component of behavioural control (i.e., H_7b_). 

### 4.3. Direct, Indirect and Total Effect Analysis

Table 5 shows the estimated direct, indirect, and total impacts of the exogenous variables on endogenous variables. On the direct effects, the predictors of attitude are relative advantage (a_11_ = 0.41), compatibility (a_21_ = 0.32), complexity (a_31_ = −0.29) and then hedonic motivation (a_41_ = 0.22). This indicates that cognitive components are stronger determinants of an attitude than the affective component. The direct predictors of behavioural control are hedonic motivation (a_42_ = 0.41), relative advantage (a_12_ = 0.38), compatibility (a_22_ = 0.33) and complexity (a_32_ = −0.25). This shows that the affective component is a stronger determinant of behavioural control as compared to cognitive components. This finding is expected because a huge part of behavioural control relates to the confidence (i.e., self-efficacy) of individuals in learning and using AVs. In this regard, possessing a positive effect has been shown to boost individuals’ confidence in using new innovations. Finally, the direct predictors for public acceptance are attitude (a_53_ = 0.61), behavioural control (a_63_ = 0.61) and subjective norms (a_73_ = 0.45).

On the indirect effects, public acceptance is most greatly influenced by relative advantage (b_13_ = 0.45). After this is compatibility (b_52_ = 0.37), hedonic motivation (b_43_ = 0.34), and complexity (b_33_ = −0.31). Figure 2 shows the impacts of the cognitive and affective components on public acceptance, fully channelled through the TPB dimensions, specifically, attitude and behavioural control. 

On the total effects, attitude has the largest total effects (c_53_ = 0.61) on public acceptance. This is followed by behavioural control (c_63_ = 0.52), relative advantage (c_13_ = 0.45), subjective norms (c_73_ = 0.43), compatibility (c_23_ = 0.37), hedonic motivation (c_43_ = 0.35) and complexity (c_13_ = −0.31). To some extent, the finding that attitude has the greatest total effect on public acceptance is consistent with previous studies, although t heir models contain different predictors which provides limited basis for comparison [5,18].

## 5. Conclusions

### 5.1. Summary 

Through the theoretical lens of TPB, the objective of this study is to recognise the factors influencing public acceptance of AVs and examine their interrelationships. This study proposes that the tenets of TPB which include attitude, subjective norms and behavioural control have direct influence on public acceptance of AVs. It further posits that attitude and behavioural control are influenced by cognitive and affective dimensions. The cognitive dimensions include relative advantage, compatibility and complexity which are obtained from the innovation diffusion framework. The affective dimension is reflected by hedonic motivation, which relates to emotions concerning the use of AVs.

A survey questionnaire was designed, and survey information was gathered from five Seoul major subway stations for a week in April 2019. In total, 525 valid responses were received. The results indicate that after accounting for control variables such as income, education and car ownership, the tenets of TPB directly explain 74% of the variance in public acceptance of AVs, which is considerably significant in the behavioural science field. Further, it has been found that the cognitive and affective dimensions have significant direct effects on attitude and behavioural control, suggesting a mediated relationship. The total effect analysis shows that attitude has the biggest impact on public acceptance. After this is behavioural control, relative advantage, subjective norms, compatibility, hedonic motivation and complexity. Overall, the findings of this study align with existing theoretical research that employs TPB to explain the public acceptance of AVs [51]. Consistent with Jing, Huang, Ran, Zhan and Shi [51], this study found that public acceptance of AVs is significantly influenced by attitude, subject norms and behavioural control. However, the ranking of the effects is different. For instance, Jing, Huang, Ran, Zhan and Shi [51] found that subjective norm has the largest effect on public acceptance whereas this study found that attitude has the largest effect on public acceptance. The difference can be explained by cultural factors such as collectivism.

### 5.2. Theoretical Contributions

This paper has value-added to academic research on AVs in several ways. Firstly, it applies TPB to study the public acceptance of AVs. As noted by Gkartzonikas and Gkritza [13], their review indicated a lack of theory-driven research to study the public acceptance of AVs despite the imminent launch of AVs in the near future. Public acceptance of AVs is crucial to achieving the critical mass for societies to realise the benefits of AVs. This study extends recent research by Acheampong and Cugurullo [25] who identified and analysed the dimensions or measurements of TPB with regards to AVs. In particular, this paper extends and contributes to the literature by examining the relationships between the dimensions of TPB and public acceptance of AVs. 

The second value-add of this study is that it has, to some extent, consolidates the literature by categorising some of the existing research using the TPB framework. For instance, research concerning individuals’ mindset about AVs is classified under the attitude category. Research regarding social influence is categorised under the subjective norms category and research relating to functional or psychological resistance is grouped under the behavioural control category. In this regard, the consolidation of the literature provides a more systematic clustering and portrayal of existing research under the TPB framework. Further, under each theme, this study has expanded the scope of the literature. For example, existing research related to subjective norms has only focused on complying with pressure from individuals’ significant referents but not on their motivation to comply with significant referents which is also an important consideration according to TPB. In addition, existing research related to behavioural control has only considered functional and psychological barriers preventing the adoption of AVs but not relating to the abilities and confidence of individuals which according to TPB, are integral components influencing the usage of AVs. These identified inadequacies have been addressed by the current study.

The third contribution of this study is that it has enriched the literature by decomposing the dimensions of TPB. The process of decomposing includes identifying and examining the determinants of TPB, which can vary according to contexts. In this study, the determinants have been proposed to include cognitive and affective components that relate to the features of AVs or outcomes of using AVs. The cognitive components are derived from the innovation diffusion theory. The theory is an appropriate theoretical lens, suitable for AVs because they are considered an innovation that will revolutionise existing transport systems. For the affective components, they are wholly reflected by hedonic motivation which is derived from the unified theory of acceptance and use of technology. To some extent, this study has contributed to a better integration of theories whereby a combination of theories has been applied and discussed to explain the public acceptance of AVs.

Finally, this study has contributed to an enhanced nomological appreciation of the factors affecting public acceptance of AVs through analysing the factors’ direct, indirect and total effects on public acceptance of AVs. It has been found that the three dimensions of TPB have direct effects on public acceptance of AVs whereas the cognitive and affective components only have indirect effects on public acceptance of AVs via attitude and behavioural control which suggests a mediated relationship. 

### 5.3. Transport Policy Implications

The findings of this study implicate a myriad of transport policies related to communication, marketing, subsidisation, infrastructure development, education and design of AVs. These policies will be elaborated in the subsequent paragraphs. Further, the findings can allow better allocation of a society’s limited resources to increase public acceptance of AVs. In particular, through this study, resources can be prioritised and dedicated to factors that are more important at improving public acceptance of AVs. 

The rankings of the determinants’ total effects on public acceptance of AVs indicate that resources should be first dedicated to improving individuals’ attitude towards AVs. Since individuals’ attitude relates to their overall assessment of AVs which can be positive or negative, the objective of policymakers is to allocate resources to ensure that the overall attitude of the public towards AVs is positive. Since AVs have not been officially launched, the public’s perception of AVs is critical to improving their attitude toward AVs. In this regard, advertising and marketing campaigns play an important role in influencing the public’s perception of AVs. Therefore, the benefits of using AVs should be widely highlighted in the campaigns to influence the attitude of the public.

The public’s attitude can be largely explained by the cognitive and affective components proposed in this study. This provides insights into the focus of the marketing and advertising campaigns. For instance, such campaigns can focus on the advantages that AVs can confer to their users as compared to conventional vehicles. In this regard, AVs can be positioned as a favourable substitute of conventional vehicles by featuring AVs’ advantages. They include enhanced safety, decreased fuel consumption, increased comfort, decreased travel time, decreased risk of impaired driving and prospects to earn extra income by employing AVs in crowdsourcing or ride-hailing services. In terms of compatibility, AVs should also be promoted as being in line with today’s values, lifestyle, historic lessons and the public’s transportation demands to improve the public’s perception of AV’s compatibility. Additionally, AVs may be aimed at working professionals, active families, and environmentally-conscious individuals who perceive AVs as more suited to their transport requirements. For complexity, simplify the process of utilising AVs by decreasing the number of AVs’ components that necessitate human involvement and the number of interactions between these components. Further decrease the AVs’ complexity by promoting the public interest, growing the public’s abilities to use AVs via tests and education, and promoting positive experiences when utilising these AVs. Finally, transport operators or policymakers can improve the hedonic motivation of users by positioning AVs to be fun and exciting. For instance, business, social or recreational functions can be incorporated in the design of AVs to encourage its acceptance and usage. 

Apart from focusing on attitude, transport policymakers can also improve individuals’ behavioural control regarding the adoption of AVs. Behavioural control possesses two dimensions: One concerns the presence of functional and psychological barriers that discourage the adoption of AVs whereas the other relates to the self-efficacy (i.e., ability and confidence) of individuals to use AVs. With regards to the first dimension, transport policymakers can offer subsidies to reduce the price of AVs when they are launched to encourage usage. Policymakers can also look into addressing AV certification, litigation and liability issues and security and privacy concerns which are noted to be major resistance preventing the adoption of AVs [4]. Furthermore, policymakers can explore ways in which AVs can be better integrated with the society’s culture and environment (e.g., ensuring the availability of customer service and interconnectivity with existing public transport network). Regarding the second dimension, policymakers can organise more education seminars, trials and training before the launch of AVs to boost users’ confidence and ability to use AVs.

Finally, since subjective norms also play a significant role in the acceptance of AVs, policymakers could actively participate in social networking and express their support towards AVs. Previous research has shown that the government’s influence and support for new initiatives or technologies are vital to garnering approval or acceptance in society. 

### 5.4. Limitations and Recommendations 

There are a few limitations in this study. For one, this research is based in Seoul, Korea, which is a city with a dense population and a high proportion of working professionals possibly in favour of the using AVs. Thus, the analysis of this research outcome needs to be done cautiously for other settings such as those in suburban, rural areas, or other countries. AVs may be advantageous in metropolitan areas similar to Seoul with significant population density because it encourages AV sharing and carpooling, enhancing the gains of employing AVs. Hence, consequent studies may contemplate studying the research model’s ability to be generalised by cross-referencing it with other situations.

A second limitation is that it has only applied TPB to understand and explain the considerations motivating public acceptance of AVs. Further study can be focused on establishing or integrating TPB with other theories or analyse the public acceptance of AVs [52].

Finally, future research can consider investigating the process of technology appropriation and vicarious reinforcement to improve the existing understanding of AV acceptance by the public. In addition, since the price or cost of using AVs would be a major consideration for individuals in their decision to use AVs, individuals’ sensitivity to the price or cost involved in using AVs compared to conventional vehicles can also be analysed in the future studies.

## Figures and Tables

**Table 1:** Constructs and indicators.

Constructs	ID	Indicator	Adapted Source
Attitude (ATD)	ATD1	I dislike (1)/like (7) the idea of using an autonomous vehicle (AV)	Taylor and Todd [41]
	ATD2	Buying an AV would be a foolish (1)/wise (7) idea	
	ATD3	I think buying an AV is a bad (1)/good (7) idea	
	ATD4	Using an AV to fulfil my daily travel needs is a bad (1)/good (7) idea	
Subjective norms (SBN)	SBN1	People who are important to me would want me to buy an AV	Moons and De Pelsmacker [23] Glanz et al. (2008)
	SBN2	People who are important to me would want me to use an AV	
	SBN3	I would comply with the demands of people who are important to me to buy an AV	
	SBN4	I would comply with the demands of people who are important to me to use an AV	
Behavioural control (BEC)	BEC1	Whether or not I use an AV is completely up to me	Han [42]
	BEC2	I have resources, time, and opportunities to use an AV	
	BEC3	I am confident that if I want, I can learn and use an AV	
Relative advantage (RLA)	RLA1	AVs would solve problems that I have encountered with conventional cars.	Jansson [43] Petschnig, et al. [44]
	RLA2	AVs would reduce the time that I need to get to places.	
	RLA3	AVs would allow better access to my intended destinations.	
	RLA4	AVs would be an environmentally friendly option.	
	RLA5	AVs would be more advantageous compared to using conventional cars.	
Compatibility (COM)	COM1	AVs would be in line with my beliefs.	Moons and De Pelsmacker [23]
	COM2	AVs would fit well with my driving habits.	
	COM3	AVs would be compatible with my mobility needs.	
	COM4	AVs would suit me well.	
	COM5	AVs would be in line with my everyday life.	
Complexity (CPL)	CPL1	AVs would be difficult to use.	Yuen, Wang, Ng and Wong [20]
	CPL2	AVs would be difficult to learn how to use.	
	CPL3	AVs would be frustrating to use.	
	CPL4	AVs would be cumbersome to use.	
	CPL5	AVs would require a lot of effort to use.	
Hedonic motivation (HMT)	HMT1	Using AVs would be fun	Venkatesh, et al. [45]
	HMT2	Using AVs would be enjoyable	
	HMT3	Using AVs would be very entertaining	
Acceptance (ACP)	ACP1	I would consider using AVs when they are available in the market.	Choi and Ji [14]
	ACP2	I would recommend AVs to my family and peers.	
	ACP3	I would encourage others to use AVs.	
	ACP4	I have positive things to say about AVs.	

Note: Except for attitude (ATD), response anchors of strongly disagree (1)/strongly agree (7) are used for each construct.

**Table 2:** Demographics of respondents.

Characteristics	Items	Frequency (*n* = 526)	Percentage (%)
Gender	Male Female	254 272	48 52
Age (years)	18 *–30 30–40 40–50 50–60 >60	186 124 86 88 42	35 24 16 17 8
Education level	Elementary or lower High school Bachelor Postgraduate	68 86 256 116	13 16 49 22
Annual income (million KRW)	<10 10–40 40–80 >80 m	116 223 147 40	22 42 28 8
Number of vehicles owned	0 1 2 >2	210 256 50 10	40 49 10 1
Driving experience (years)	No license <1 1–5 5–10 >10	101 56 133 156 80	19 11 25 30 15

* The legal age for driving a vehicle is 18 years.

**Table 3:** Confirmatory factor analysis.

Construct	Indicator	λ	AVE	CR
Attitude (ATD)	ATD1 ATD2 ATD3 ATD4	0.84 0.89 0.91 0.86	0.77	0.93
Subjective norms (SBN)	SBN1 SBN2 SBN3 SBN4	0.87 0.86 0.84 0.89	0.75	0.92
Behavioural control (BEC)	BEC1 BEC2 BEC3	0.92 0.94 0.93	0.87	0.95
Relative advantage (RLA)	RLA1 RLA2 RLA3 RLA4 RLA5	0.72 0.76 0.84 0.88 0.82	0.65	0.90
Subjective norms (SBN)	COM1 COM2 COM3 COM4 COM5	0.73 0.86 0.95 0.91 0.80	0.73	0.93
Complexity (CPL)	CPL1 CPL2 CPL3 CPL4 CPL5	0.86 0.84 0.76 0.72 0.88	0.66	0.91
Hedonic motivation (HMT)	HMT1 HMT2 HMT3	0.79 0.87 0.89	0.72	0.89
Acceptance(ACP)	ACP1 ACP2 ACP3 ACP4	0.92 0.88 0.93 0.90	0.82	0.95

Note: Model fit indices: *χ*^2^/df = 1.98 (*p* < 0.05); Comparative Fit Index(CFI) = 0.97; Tucker-Lewis Index (TLI) = 0.98; Root Mean Square Error of Approximation (RMSEA) = 0.06; Standardised Root Mean Residual (SRMR) = 0.08.

**Table 4:** AVE, correlations, and squared correlations of the constructs.

	ATD	SBN	BEC	RLA	COM	CPL	HMT	ACP
ATD	**0.77**	0.19	0.03	0.03	0.17	0.38	0.05	0.02
SBN	0.23	**0.75**	0.02	0.01	0.35	0.21	0.01	0.03
BEC	0.15	0.13	**0.87**	0.01	0.07	0.07	0.05	0.03
RLA	0.46	0.05	0.42	**0.65**	0.24	0.10	0.04	0.03
COM	0.42	0.03	0.39	0.44	**0.73**	0.38	0.04	0.06
CPL	–0.38	–0.07	–0.32	–0.16	–0.15	**0.66**	0.52	0.26
HMT	0.32	0.10	0.49	0.08	0.07	–0.08	**0.72**	0.42
ACP	0.73	0.56	0.63	0.07	0.10	–0.10	0.09	**0.82**

Note: Main diagonal reflects AVE values; Values below the main diagonal are correlations; Constructs above the main diagonal are squared correlations.

**Table 5:** Direct, indirect and total effects.

	Endogenous (j)	Attitude (1)	Behavioural Control (2)	Public Acceptance (3)
Exogenous (i)				
Direct effects (aij) of …				
relative advantage (1)		0.41	0.38	—
compatibility (2)		0.32	0.33	—
complexity (3)		−0.29	−0.25	—
hedonic motivation (4)		0.22	0.41	—
attitude (5)		—	—	0.61
behavioural control (6)		—	—	0.52
subjective norms (7)		—	—	0.43
Indirect effects (bij) of …				
relative advantage (1)		—	—	0.45
compatibility (2)		—	—	0.37
complexity (3)		—	—	−0.31
hedonic motivation (4)		—	—	0.35
attitude (5)		—	—	—
behavioural control (6)		—	—	—
subjective norms (7)		—	—	—
Total effects (cij) of …				
relative advantage (1)		0.41	0.38	0.45
compatibility (2)		0.32	0.33	0.37
complexity (3)		−0.29	−0.25	−0.31
hedonic motivation (4)		0.22	0.41	0.35
attitude (5)		—	—	0.61
behavioural control (6)		—	—	0.52
subjective norms (7)		—	—	0.43
